# Stabilization of PE with Pomegranate Extract: Contradictions and Possible Mechanisms

**DOI:** 10.3390/antiox11020418

**Published:** 2022-02-18

**Authors:** Dóra Tátraaljai, Yun Tang, Emese Pregi, Erika Vági, Viola Horváth, Béla Pukánszky

**Affiliations:** 1 Institute of Materials and Environmental Chemistry, Research Centre for Natural Sciences, ELKH Eötvös Lóránd Research Network, P.O. Box 286, H-1519 Budapest, Hungary; pregi.emese@vbk.bme.hu (E.P.); pukanszky.bela@vbk.bme.hu (B.P.); 2 Laboratory of Plastics and Rubber Technology, Department of Physical Chemistry and Materials Science, Budapest University of Technology and Economics, P.O. Box 91, H-1521 Budapest, Hungary; 3 Department of Chemical and Environmental Process Engineering, Budapest University of Technology and Economics, P.O. Box 91, H-1521 Budapest, Hungary; tangyun0331@163.com (Y.T.); vagi.erika.maria@vbk.bme.hu (E.V.); 4 Department of Inorganic and Analytical Chemistry, Budapest University of Technology and Economics, P.O. Box 91, H-1521 Budapest, Hungary; horvath.viola@vbk.bme.hu; 5 MTA-BME Computation Driven Chemistry Research Group, P.O. Box 91, H-1521 Budapest, Hungary

**Keywords:** natural antioxidants, natural extract, pomegranate, stabilization, long chain branching, mechanism

## Abstract

Dry pomegranate peel was extracted with acetone and the extract was added to a Phillips type polyethylene. The concentration of the extract was changed from 0 to 1000 ppm in six steps and stabilization efficiency was checked by the multiple extrusion of the polymer followed by the characterization of chemical structure, processing, and residual stability. The results confirmed the excellent processing stabilization efficiency of the extract, but also the poor long-term stability of PE containing it in accordance with previously published results. The extract is amorphous and its solubility is relatively large in the polymer; thus, these factors cannot be the reason for the poor stabilization efficiency in an oxygen-rich environment. Chemical factors like the self-interaction of the polyphenol molecules, the stability of the radicals forming after hydrogen abstraction, and the lack of hydrogens with the necessary reactivity must be considered during the evaluation of the efficiency of the extract. These factors as well as the insufficient number of active hydrogens hinder the reaction of the additive molecules with oxygen-centered radicals, thus leading to inferior long-term stability. The extract can be used for the processing stabilization of polymers, but for applications requiring long-term stability, it must be combined with other natural antioxidants like flavonoids or Vitamin E.

## 1. Introduction

Polyethylene (PE) is a commodity polymer produced and used in the largest quantity in the world. During its processing and use, it is subjected to heat, shear, and oxygen, resulting in chemical reactions. The reactions change the structure of the polymer, i.e., it degrades, and thus its properties deteriorate. Accordingly, the polymer must be protected against degradation to maintain its properties during processing and application. Various additive packages are used for the stabilization of polyethylene, depending on the application of the product. These packages usually contain at least one hindered phenolic antioxidant and a secondary stabilizer, usually a phosphorous or sulfur containing compound, but possibly many other components, such as light stabilizers, colorants, lubricants, antifungal agents, etc. The use of a phenolic primary and a phosphorous secondary antioxidant is a well-established industrial practice, but recently more and more attempts are being made to replace phenolic antioxidants with naturally occurring ones. Quite a few reports have been published recently on the use of natural compounds for the stabilization of polyethylene; various flavonoids [1,2,3,4,5], carotene [6], Vitamin E [7,8,9], and lignin [10,11,12] were used with small or large success for stabilization. Today, Vitamin E is routinely used for the stabilization of medical devices, as well as in the food industry [7,8,9].

Most of the experiments aimed at the determination of the stabilization effect and efficiency of natural substances are usually conducted on pure compounds extracted from plants and then separated to obtain a material with high purity. However, separation is tedious and expensive; thus, more and more attempts are being made to use natural extracts, as obtained from the plant. A good candidate for stabilization is the extract of pomegranate, which contains numerous components with antioxidant effects. Pomegranate is a popular fruit that is grown in large quantities in tropical and subtropical areas, mainly in Asia. The edible part of the fruit is less than 50% and the rest is waste. However, all parts of the fruit contain useful components [13,14]. Its peels are the source of phenolics, minerals, and complex polysaccharides, while arils contain sugars, pectin, organic acids, phenolics, and flavonoids. Large amounts of proteins, crude fibers, vitamins, minerals, pectin, sugars, polyphenols, and isoflavones can be found in seeds, and their oil contains linolenic and linoleic acids, as well as other lipids such as punicic, oleic, stearic, and palmitic acid [15,16].

Various parts of the fruit have been used in traditional medicine for ages. Diarrhea and dysentery are traditionally cured by the peels of pomegranate, but most parts of the fruit have proven antioxidant, anti-carcinogenic, and anti-inflammatory effects [17,18,19]. Because of the large quantity of waste formed during the consumption and processing of the fruit, interest in the use of the extract of pomegranate components, especially the peels, is increasing. Pomegranate peel extract is successfully used in the food industry for conservation and to increase the quality of food. It can be used also as an antioxidant, colorant, and flavor-enhancer [13]. However, we are aware of only two papers on the use of pomegranate peel extract for the stabilization of polyethylene. Xia et al. [20] compared the effect of an ethanol extract of pomegranate peel to that of the hindered phenolic antioxidant used in the largest quantity in industrial practice. They found that the extract is a good overall stabilizer, which protects the polymer during processing, against oxidation, and UV radiation, and it is at least as efficient as the phenol. Tátraaljai et al. [21], on the other hand, found that although the extract protected PE very efficiently during processing, the long-term stability of the polymer was poor. In this latter case, active components were extracted from pomegranate peel by acetone by the researchers themselves. The findings of Xia et al. [20] and Tátraaljai et al. [21] obviously contradict each other on the efficiency of the extract as an antioxidant in an oxygen-rich environment.

Previous results unambiguously show that pomegranate peel extract is a potential stabilizer for polymers [20,21]. However, as mentioned above, the existing information is insufficient and contradictory; further research is needed to establish the full potential of the substance. In our previous study, we merely wanted to establish the possibility to use the POM extract as a stabilizer and investigated its performance at a single concentration in comparison with industrial stabilizer packages [21]. On the other hand, the goal of this study was to obtain more information on the stabilization effect and efficiency of pomegranate peel extract. Accordingly, here we discuss the composition of the POM extract in detail, analyze the controversy mentioned, study stabilization as a function of additive content, and consider several additional aspects of stabilization, including interactions and solubility of the extract in the polymer. An attempt is made to resolve the contradiction mentioned above and develop some ideas about the mechanism of stabilization. We used the results obtained in our earlier research as reference [4,21]. Conclusions and possible relevance for practice are briefly discussed in the final section of the paper.

## 2. Materials and Methods

### 2.1. Materials

The polymer used in the experiments was the Tipelin FS 471 grade ethylene/1-hexene copolymer (melt flow rate: 0.3 g/10 min at 190 °C, 2.16 kg; nominal density: 0.947 g/cm^3^) polymerized with a Phillips catalyst. The additive free polymer powder was provided by MOL Group Ltd. (Tiszaújváros, Hungary). The extract used for stabilization was prepared from dried pomegranate peel powder (Turkey). Soxhlet extraction with acetone as solvent was used to obtain the active components, which was carried out until complete exhaustion of the powder (7 h daily for 4–5 days). Acetone was removed by vacuum distillation. A yellow, lightly scented powder was obtained at the end of the process. Punicalagin A + B (97%) and ellagic acid (99%) standards were purchased from PhytoLab GmbH & Co., KG (Vestenbergsgreuth, Germany) for the quantitative determination of these components in the extract. HPLC gradient grade solvents (acetonitrile, ethanol) were used in the analysis, which were purchased from Merck KGaA (Darmstadt, Germany). 2,2-diphenyl-1-picrylhydrazyl (DPPH) free radical was purchased from Merck for the DPPH assay. Folin & Ciocalteu’s phenol reagent (2 N) and sodium carbonate (anhydrous, ACS reagent > 99.5%) were also obtained from Merck. Pyrogallol (>98% HPLC) standard, used for the measurement of total polyphenol content, was purchased from Honeywell-Fluka (Charlotte, NC, USA). Ninety-six percent ethanol and methanol were purchased from Molar Chemicals Ltd. (Halásztelek, Hungary). The pomegranate peel extract (POM) was added to the polymer at 50, 100, 250, 500, 1000 ppm in combination with the 1000 ppm Sandostab PEPQ (PEPQ, Clariant, Basel, Switzerland) secondary stabilizer. The effect of 1000 ppm PEPQ alone was also determined for comparison. The polymer was also stabilized by 1000 ppm Irganox 1010 (I1010, BASF, Nienburg/Weser, Germany) in combination with 1000 ppm PEPQ for comparative purposes and the stabilization effect of POM alone was also determined in the polyethylene used.

### 2.2. Sample Preparation

The additives were mixed with PE powder in a Henschel FM/A10 high-speed mixer (Thyssen, Kassel, Germany) at the rate of 1000 rpm for 10 min. The POM extract was added to the powder in the form of acetone solution to provide uniform distribution. The acetone was removed by storage at ambient temperature. The dry mixture was processed and pelletized in six consecutive extrusion steps at 50 rpm and at the barrel temperatures of 180, 220, 260, and 260 °C using a Rheomex S ¾” (Haake, Saddle Brook, NJ, USA) type single screw extruder attached to a Rheocord EU 10V (Haake, Saddle Brook, NJ, USA) driving unit. Samples were taken after each step. Films of about 100 µm thickness were prepared by compression molding for further studies at 190 °C and 5 min (3 min preheating and 2 min compression time) using a Fontijne SRA 100 (Vlaardingen, The Netherlands) machine.

### 2.3. Characterization

The antioxidant activity of pomegranate peel extract and I1010 were evaluated by the DPPH assay [22]. Three parallel measurements were carried out for each concentration in methanol solution using a Camspec M501 (Leeds, UK) spectrophotometer. Total polyphenol content was determined by a spectrophotometric method using Folin-Ciocalteu reagent and pyrogallol as reference standard. Concentration was determined from the absorbance appearing at 760 nm using the same Camspec M501 spectrophotometer [23]. The crystallinity of the extract and I1010 was determined by a Perkin Elmer DSC 7 (Norwalk, CT, USA) apparatus. 3–5 mg samples were heated from 30 to 300 °C at 10 °C/min heating rate in nitrogen atmosphere. The concentration of punicalagin and ellagic acid was determined using a Perkin Elmer Series 200 HPLC (Norwalk, CT, USA) apparatus equipped with a diode array detector. A reversed phase YMC-Pack ODS-AQ C18 (150 × 4.6 mm; 3 µm) column (Kyoto, Japan) was applied for the separation. The mobile phase solvent A was 0.1% phosphoric acid in water and solvent B was acetonitrile. The following gradient was applied: solvent B % was increased from 2% to 9.5% within 15 min, then from 9.5% to 20% within 42 min, followed by an increase from 20% to 25% in 10 min. A step gradient at 80% B for 5 min was used to elute the compounds with long retention times, and finally, re-equilibration at the initial concentration of solvent 2% B took place in 10 min. The melt flow rate (MFR) of the polymer was determined according to the ASTM D 1238-79 standard at 190 °C with 2.16 kg load using a Göttfert MPS-D (Buchen, Germany) MFR tester. The measurements were repeated five times in each case. Residual thermo-oxidative stability was characterized by the oxidation induction time (OIT). The measurements were done in oxygen atmosphere at constant, 20 mL/min flow rate, and 190 °C in triplicates. Opened aluminum pans and the same Perkin Elmer DSC 7 (Norwalk, CT, USA) apparatus were used according to the ISO 11357-6:2018 standard. The concentration of the unsaturated functional groups of polyethylene was determined by Fourier-transform infrared spectroscopy (FTIR) on the 100 μm thick compression molded films in transmission mode using a Tensor 27 (Bruker Optik GmbH, Leipzig, Germany) spectrophotometer. Five parallel measurements were carried out on each sample between 4000 and 400 cm^−1^ wavelengths at 2 cm^−1^ resolution by 16 scans. The concentration of vinyl groups was calculated from the intensity of the absorption peak appearing at 908 cm^−1^, while vinylidene concentration was determined from the absorption band at 888 cm^−1^. Carbonyl content was calculated from the integrated area determined between 1778 and 1691 cm^−1^. FTIR spectroscopy was used also for the determination of residual PEPQ content based on the absorption of the P(III)-O-C groups at 850 cm^−1^. The detailed calculation of the above-mentioned concentrations was given in our earlier publication [21]. The chemical structure of the additives was also studied by FTIR spectroscopy using the same Tensor 27 apparatus in the Attenuated Total Reflectance (ATR) mode. A Hunterlab ColorQuest 45/0 (Reston, VA, USA) apparatus was used for the determination of the yellowness index (YI) of the polymer samples in triplicates.

## 3. Results

The results are presented in several sections. First, an overview is offered about the components and composition of POM extract and then the controversial results related to its use as stabilizer for PE are discussed briefly. The dependence of PE properties on the concentration of POM is analyzed next. Factors other than chemical composition are discussed in the next section, while considerations about effect, efficiency, and stabilization mechanism are presented in the last section of the paper.

### 3.1. Composition, Main Components, and Activity

Pomegranate peel extract contains numerous components. A research group [15] found that pomegranate peel contained 79 phenolic compounds, including 16 phenolic acids, 12 flavonoids, 35 hydrolyzable tannins, 8 proanthocyanidins, and 8 anthocyanins. Fischer et al. [16], on the other hand, analyzed extracts and juices of pomegranate by HPLC-DAD–ESI/MS^n^ (high-performance liquid chromatography coupled to electrospray ionization mass spectrometric, detection in the negative ion mode) and found 48 compounds in them. Accordingly, the complete identification and especially the quantitative analysis of all components of pomegranate extracts are rather challenging, because components and quantities depend on many factors, including location, source, extraction technique, etc.

In order to give some idea about the complexity of the analysis of extracts prepared from the various parts of pomegranate, an overview is offered in Table 1, showing the extraction method, the three main components identified by the respective group and their amount, if determined.

**Figure 1:**
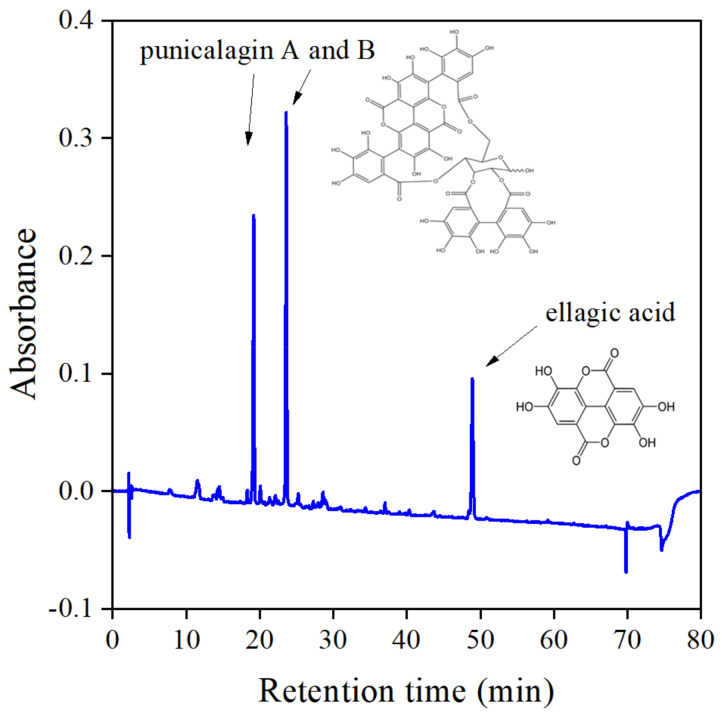
HPLC chromatogram recorded on the acetone extract of pomegranate peel. The main components are punicalagin and ellagic acid.

According to the table, mostly alcohols and water, as well as their combination, are used for extraction. Acetone is used occasionally, but we found that components with large antioxidant activity can be extracted with this solvent the most advantageously. Although components and composition vary in a wide range, basically all sources agree that the main components of the peel are punicalagin, granatin, ellagic, and gallic acid. The amounts determined are even more controversial than the number and type of components. Punicalagin content varies from 2 to 500 mg/g dry peel, depending on the extraction method and the source of the fruit. The findings of Rongai et al. [31] are especially interesting—they investigated a number of fruits from different areas and sources and found punicalagin content to change between 1.6 and 476.0 mg/g. The composition and thus the efficiency of the extract as stabilizer obviously depends on a number of factors. Nevertheless, our findings agree well with those listed in Table 1. As Figure 1 shows, three main components were found by HPLC analysis, which were identified as punicalagin A and B, as well as ellagic acid. Small amounts of other components were also detected, but could not be identified unambiguously.

**Scheme 1:**
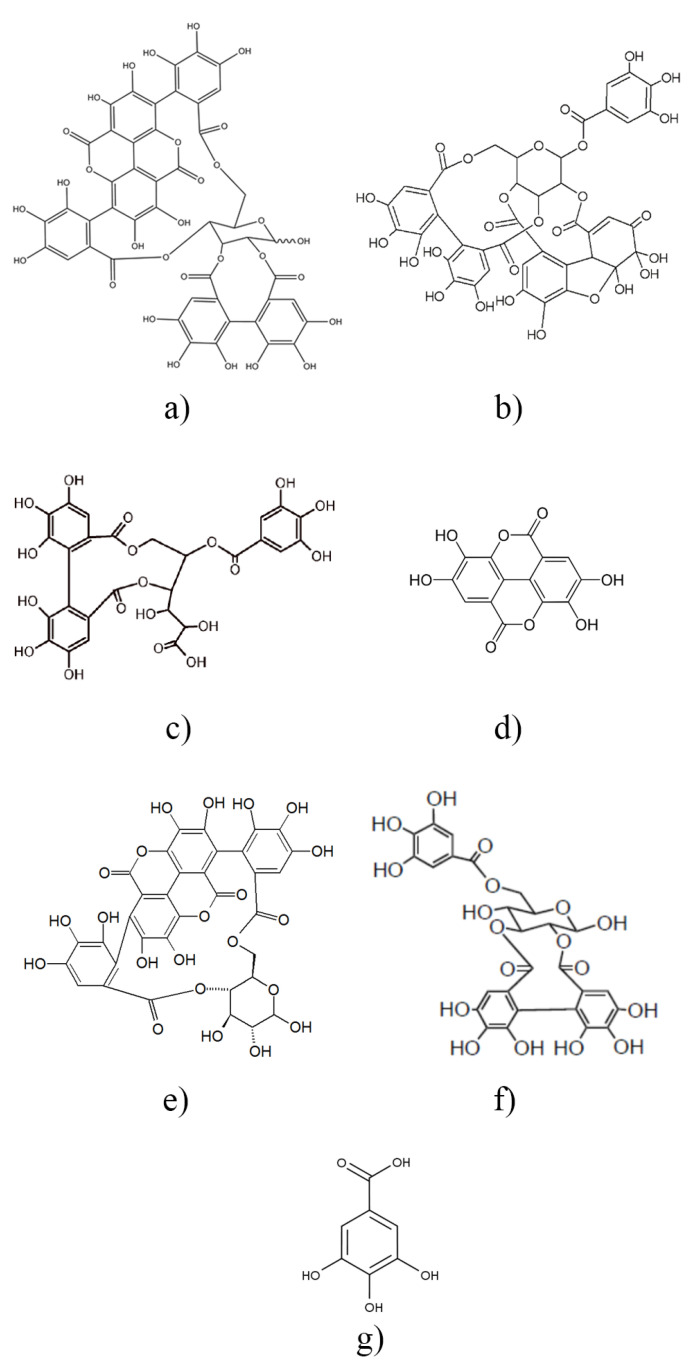
Chemical structure of the main components of pomegranate peel extracts listed in Table 1. punicalagin (**a**), granatin B (**b**), galloyl-HHDP-glucose (**c**), ellagic acid (**d**), punicalin (**e**), galloyl-HHDP-hexoside (**f**), gallic acid (**g**).

**Figure 2:**
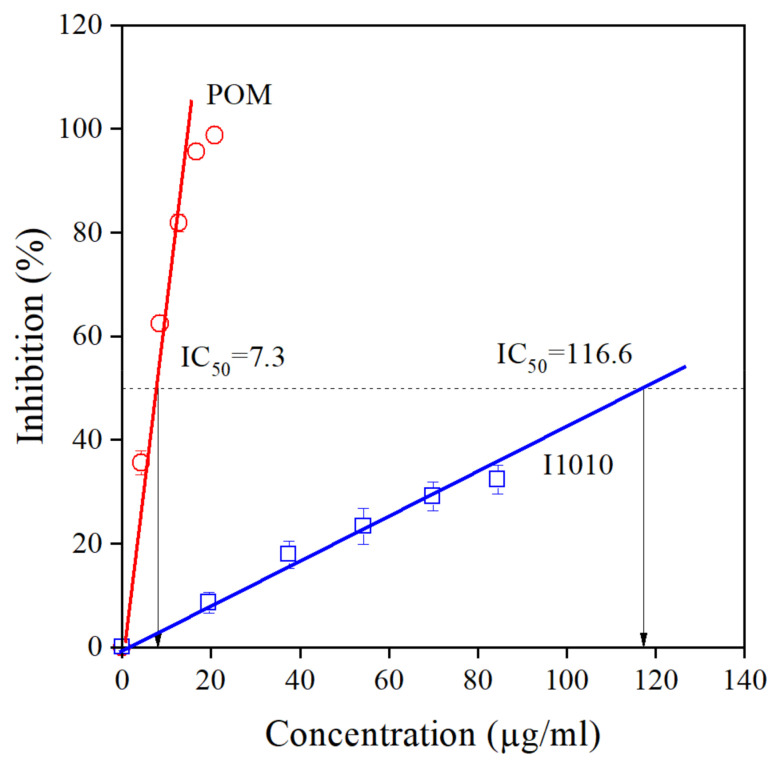
Determination of the antioxidant activity of the POM extract and the commercial hindered phenolic antioxidant I1010 by DPPH assay. Symbols: (○) POM, (□) I1010.

The chemical structure of all compounds listed in Table 1 is presented in Scheme 1. According to the scheme, all components contain phenolic OH groups—some of them have several such moieties. Phenolic hydroxyl groups are responsible for the antioxidant activity of the compounds; thus, it is not very surprising that basically all research groups found strong antioxidant effect for their extracts irrespectively of source or extraction method. The antioxidant activity of the extract prepared and studied in this project was determined by the DPPH assay approach. The DPPH assay expresses activity by the EC_50_ (Effective Concentration) or IC_50_ (Inhibition Concentration) value, which is defined as the concentration of the substrate that results in a 50% loss in DPPH activity [22]. Accordingly, the number determined is reversibly proportional to the antioxidant activity of the compound—the smaller the value, the larger the activity. The result of the determination is presented in Figure 2. The activity of the commercial hindered phenolic antioxidant used as reference was also determined for comparison. The results clearly prove that the POM extract prepared and used in this study is much more effective in the DPPH assay than the commercial stabilizer and consequently we should expect a much stronger stabilization effect as well. According to our own measurements, the total phenolic content of POM is 32.50 ± 0.16 g pyrogallol equivalent/100 g extract, while for the I1010, this number is just 0.83 ± 0.04 g/100 g.

### 3.2. The Controversy

As mentioned earlier, only two reports are available on the effect of pomegranate extract on the stabilization of PE, according to our knowledge. Xia et al. [20] used an ethanol extract and found it to be an excellent overall stabilizer, including processing, oxidative, and light stabilization. They used the extract at 0.12 and 0.7 wt% with and without a secondary antioxidant, PEPQ, and compared the effect to that of 0.12 wt% of Irganox 1010. They also investigated the extract alone at 2 wt%, i.e., 20,000 ppm concentration, which is much larger than the amount applied usually. They characterized oxidative stability by the oxidation onset temperature (OOT), which is used frequently, but according to our experience, it is less informative on residual stability than OIT.

**Figure 3:**
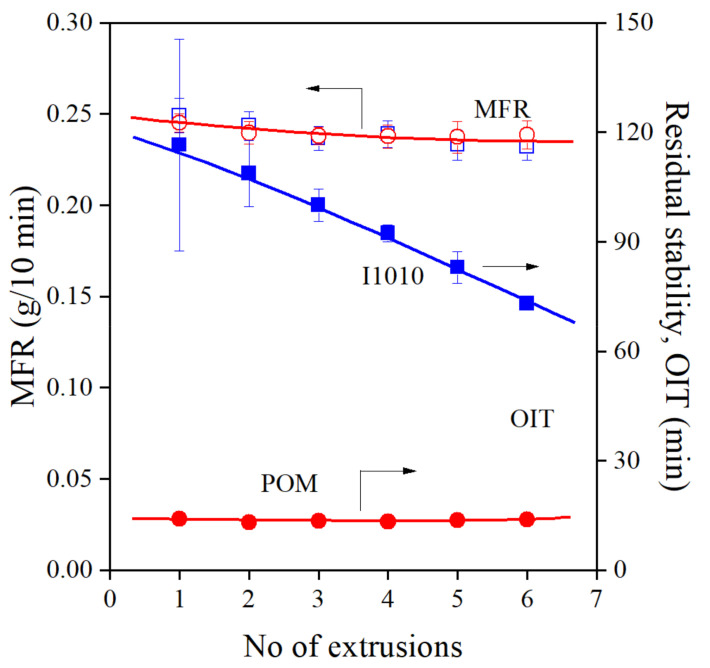
Comparison of the melt (MFR) and long-term (OIT) stabilization efficiency of the POM extract and I1010 at 1000 ppm antioxidant and 1000 ppm PEPQ content. (Summary of our previous results [21].) Symbols: (○,●) POM, (□,■) I1010; empty symbols: MFR, full symbols OIT.

The results of Tátraaljai et al. [21] do not agree completely with those of the Chinese group. Although the efficiency of the POM extract was excellent in processing stabilization, the residual stability of the PE compounds prepared was very small. The controversy is demonstrated quite well by Figure 3, in which the changes in MFR, indicating processing stabilization and residual stability are plotted against the number of extrusion steps. MFR and OIT values were taken from our previous study [21] to demonstrate the controversy discussed. Although the POM extract had the same efficiency in processing stabilization as I1010, residual stability was much smaller in its presence.

The results available contradict each other on several points. The DPPH assay indicated much larger activity for POM than for I1010. The processing stabilization effect of the two additives is approximately the same, as shown by Figure 3. Xia et al. [20] found good overall stabilization effect, including oxidative stability, but according to Tátraaljai et al. [21], residual stability is small. Finally, the very small OIT contradicts also the strong antioxidant activity of the extract (DPPH assay) and the large total phenolic content. Obviously, it can trap carbon center radicals during processing, but cannot react with oxygen centered species in the oxygen-rich environment of the OIT measurement. Additionally, Xia et al. [20] claim that the extract can be used with large efficiency also without a phosphorous secondary antioxidant, although the use of 20,000 ppm of the substance makes this claim somewhat doubtful. The results available up to now raise a number of questions and strongly justify further experiments.

### 3.3. Composition Dependence

**Figure 4:**
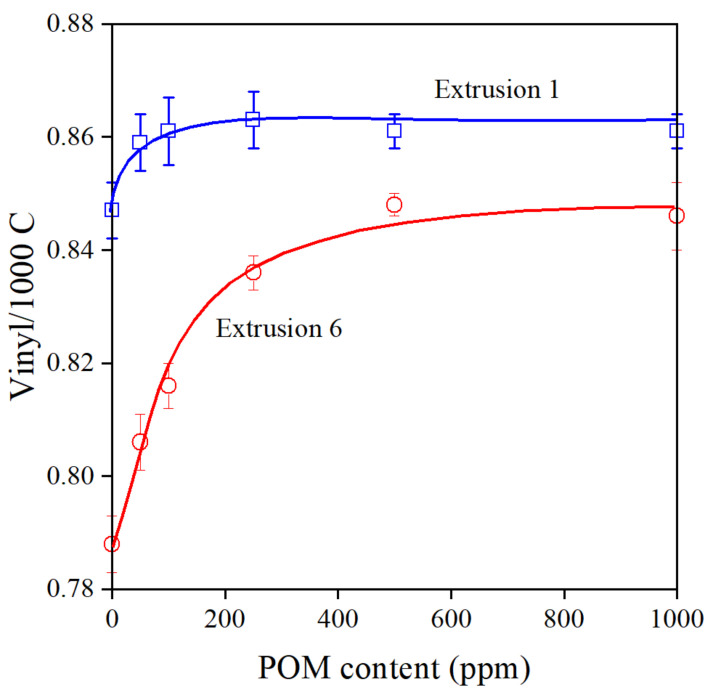
Composition dependence of the vinyl group content of PE stabilized with the POM extract after the first and sixth extrusion. Symbols: (○) 1st, (□) 6th extrusion.

As mentioned earlier, reactions take place during the processing of polymers, which lead to the modification of their structure. Stabilizers modify the reactions and protect the polymer against any undesirable change of structure. The effect obviously depends on the composition of the additive package and the concentration of the stabilizer. In the case of the Phillips PE used in this study, the main degradation reaction is the addition of alkyl radicals to vinyl groups located at one end of each polymer chain [33,34,35]. The vinyl group content of the polymer is plotted against the concentration of the pomegranate peel extract after the first and sixth extrusion in Figure 4. As expected [21], the POM extract protects the polymer very efficiently even at small concentrations, and vinyl content becomes constant between 100 and 200 ppm at least after the first extrusion. The decrease of vinyl content is significant after six extrusion steps in the polymer without POM, and at least 500 ppm POM extract is needed to block all reactions leading to long chain branching; however, we can safely conclude that the extract is indeed a very efficient processing stabilizer for PE.

**Figure 5:**
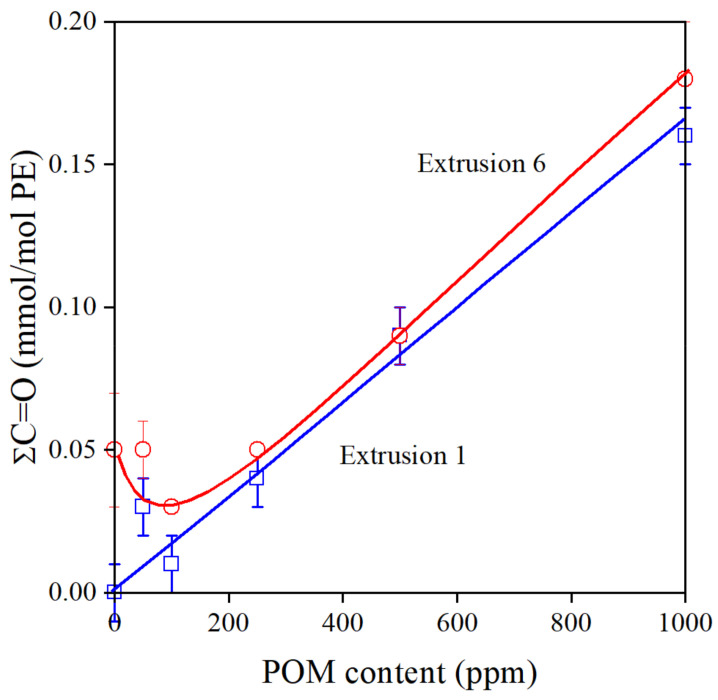
Effect of POM content and processing history on the total carbonyl content of PE stabilized with the natural extract. Symbols: (○) 1st, (□) 6th extrusion.

Polymers always contain some dissolved oxygen and oxygen is also absorbed on the surface of the raw material [36]. Oxidation leads to the formation of carbonyl groups [37,38]. The overall carbonyl content of the polymer is plotted against POM content in Figure 5. The number of this functional group increases linearly after the first extrusion, and following a small decrease; it shows the same tendency after the sixth processing step. If we check the structure of the most important components of the pomegranate extract, we can see that most of them contain carbonyl groups inherently (see Scheme 1); thus, it is not very surprising that carbonyl content increases with the concentration of the additive. The small decrease at small additive content after the 6th extrusion indicates that oxidation takes place and additional carbonyl groups form, but at larger concentrations, above 100–200 ppm, these reactions are hindered efficiently.

**Figure 6:**
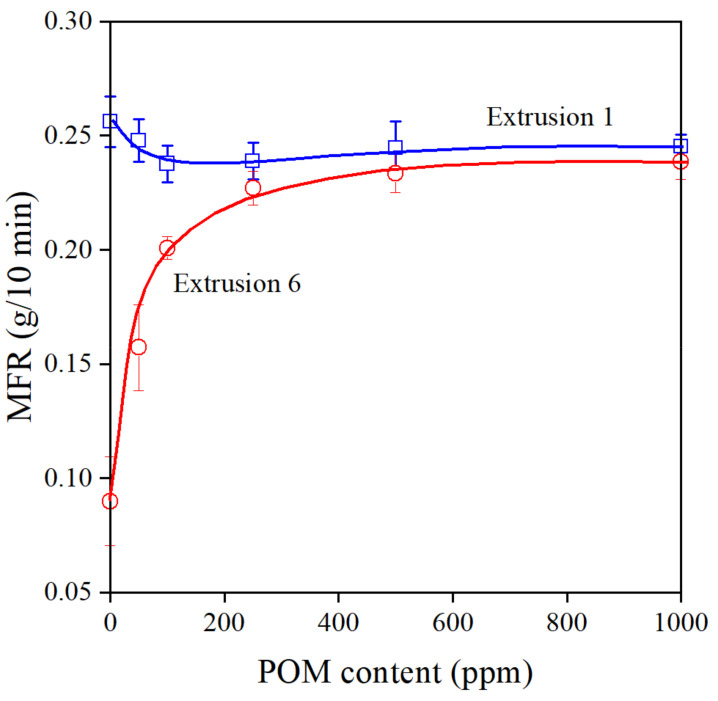
Changes in the viscosity (MFR) of PE containing various amounts of POM extract. Symbols: (○) 1st, (□) 6th extrusion.

Changes in the melt flow rate of the polymer indicate very sensitively the efficiency of stabilizers during processing. The formation of long chain branches increases viscosity, which leads to the decrease of MFR. The effect of the amount of POM extract on MFR is shown in Figure 6. The extract is a very efficient processing stabilizer indeed; MFR increases steeply after the sixth extrusion and reaches a constant value at around 500 ppm additive content. The small decrease of MFR with increasing additive content in the first extrusion step might be surprising, since a larger amount of stabilizer should lead to larger MFR. However, we must consider the structure of the compounds constituting the majority of the extract. They are all very polar, containing groups that are capable of forming hydrogen bonds; thus, they will definitely interact with each other in the very apolar medium of the polyethylene melt. At smaller concentrations, these interactions decrease the efficiency of the stabilizer and lead to a slight decrease in melt flow rate.

**Figure 7:**
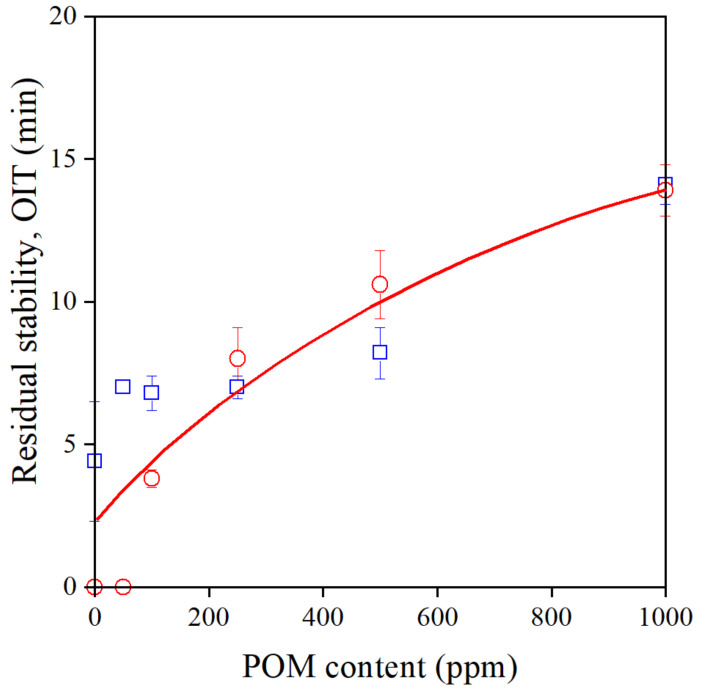
The residual stability (OIT) of Phillips PE plotted against the POM extract content of the polymer. Symbols: (○) 1st, (□) 6th extrusion.

The POM extract proved to be a very efficient processing stabilizer, as shown above. On the other hand, the residual stability of the polymer was surprisingly small in its presence (see Figure 3) even when it was added at 1000 ppm, along with a secondary stabilizer. Residual stability, i.e., OIT, is plotted against POM content in Figure 7. Although OIT increases with increasing additive content, it remains quite small even at 1000 ppm, as observed before. Quite surprisingly, there is hardly any difference in the stability of samples extruded once or six times, indicating that the efficiency of the extract in preventing degradation in an oxygen-rich environment of the OIT measurement is extremely small. These experiments have confirmed again that the POM extract is a good processing stabilizer, but does not provide long-term stability for the polymer. Further considerations are needed to resolve this controversy.

### 3.4. Structure, Solubility, Interactions

**Figure 8:**
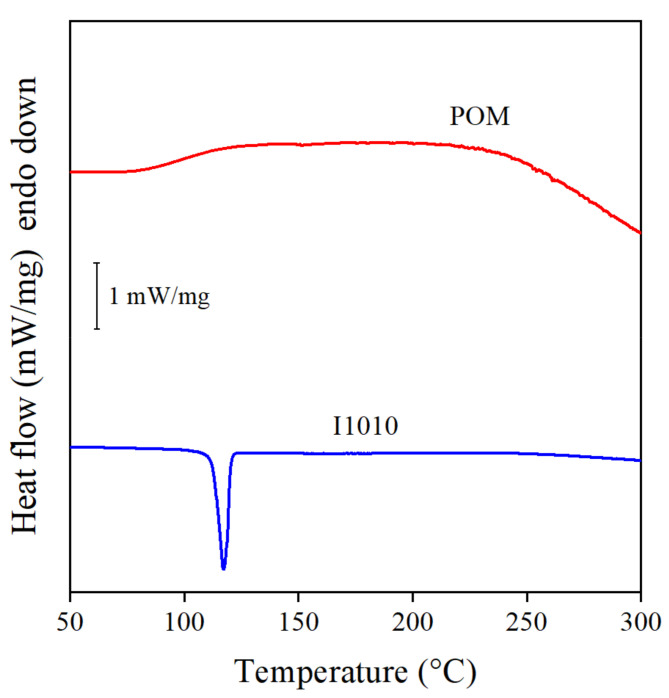
DSC curves recorded on the neat POM and I1010 additives to determine crystallinity and melting temperature.

Besides chemical structure, the efficiency of stabilizers also depends on other factors. One of them is crystallinity and the melting temperature of the crystals. Quercetin, for example is a very efficient natural antioxidant, but it has a very high melting temperature, 316 °C, which is higher than the usual processing temperature of PE or even the high temperatures used in this study to determine the efficiency of the stabilizer. This high melting temperature makes handling and homogenization very difficult [4]. Crystalline substances do not dissolve in PE, because the weak interactions acting in the polymer are not sufficient to melt and distribute at the molecular level [39]. POM is a natural compound with components similar in structure to quercetin; thus, it also could be crystalline. This possibility had to be checked to determine its effect on dissolution and stabilization efficiency, since crystallinity reduces the solubility of the antioxidant and its efficiency as a stabilizer in the polymer matrix [39]. Ordered structure, i.e., crystallinity, can be conveniently determined by DSC measurements. The DSC traces of the two additives, the extract and I1010 used as reference, are presented in Figure 8. The sharp melting peak of the commercial stabilizer clearly proves its crystalline nature. The POM extract, on the other hand, is completely amorphous, no melting peak can be observed on the DSC curve at all. Obviously, crystallinity cannot be the reason for the inefficiency in providing sufficient residual stability.

**Figure 9:**
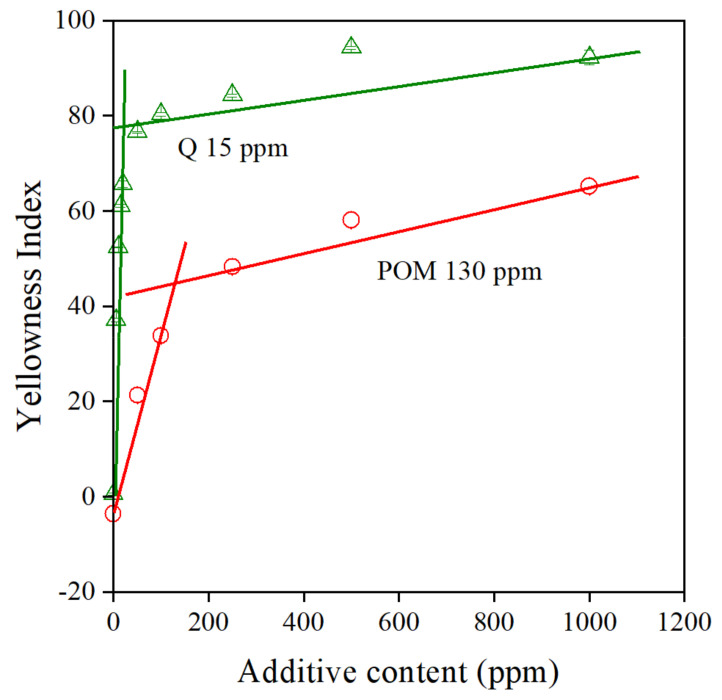
Determination of the solubility of additives from the composition dependence of the color of PE compounds. Quercetin (Q) was used as reference. Symbols: (△) quercetin, (○) POM extract.

The solubility of the additive in the matrix polymer is another important issue. Stabilizers are usually polar compounds, while polyethylene is completely apolar—it does not contain any functional groups. Accordingly, the solubility of most additives is very small in PE. The solubility of colored additives can be determined reasonably easily from the color of the polymer. According to Lambert–Beer’s law, the latter should increase linearly with the concentration of the additive up to the solubility limit, until the mixture is homogeneous [40]. The increase in color is much slower when the stabilizer forms a separate phase and the diameter of the coloring substance increases [41]. We discussed this phenomenon and proved the formation of a separate phase by the antioxidant with digital optical microscopy in one of our previous papers [4]. The color of PE containing POM is plotted against stabilizer content in Figure 9. The correlations clearly show that the concept works—solubility can be determined by this approach. Quercetin (Q), another natural antioxidant, is used as reference here for comparison. The results were taken from one of our earlier works [4], as mentioned above. The solubility of this latter compound is very small—15 ppm. On the other hand, the solubility of the POM extract is surprisingly large, almost an order of magnitude larger than that of quercetin, 130 ppm. Larger solubility should lead to better homogeneity and larger efficiency, which in fact is proved by the excellent processing stabilization efficiency of the extract.

**Figure 10:**
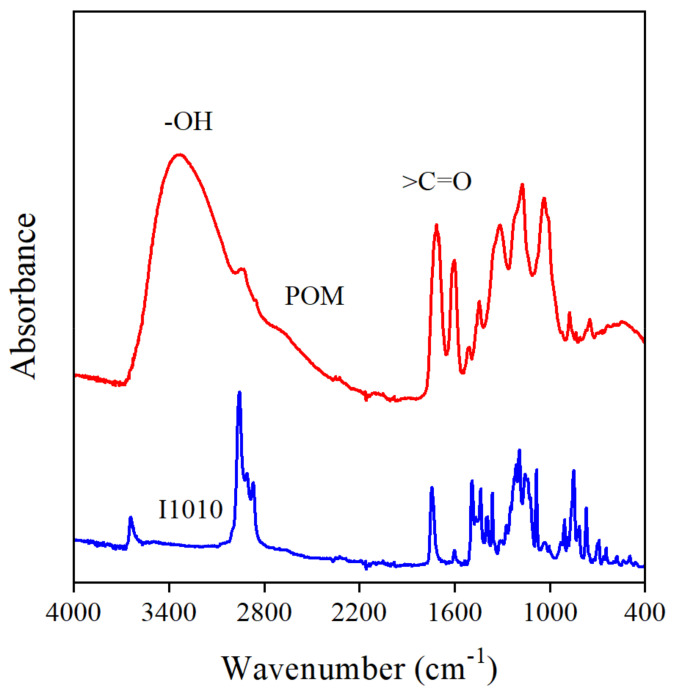
FTIR spectra of POM and I1010, respectively. The broad absorbance in the 3700–3000 cm^−1^ region indicates the interaction of polyphenol molecules.

The interaction of the additive molecules was mentioned above as a possible reason for the decrease in MFR at small POM contents (see Figure 6). The POM extract contains a large number of functional groups, hydroxyl, carbonyl, ether, lactone, etc., which are capable of forming interactions with each other. The FTIR spectra of POM and I1010 are presented in Figure 10. Carbonyl groups can be detected clearly in both additives, but more interesting for us is the region above 3000 cm^−1^. On the spectrum of I1010, a small sharp peak at 3640 cm^−1^ indicates the presence of the phenolic OH groups of the stabilizer, which do not interact with each other. On the other hand, a very broad absorbance band can be detected in the spectrum of the POM extract, which can be assigned to the absorbance of hydrogen-bonded OH groups, indicating that the molecules of the compounds in the extract strongly interact with each other indeed. This interaction definitely competes with stabilization reactions and decreases the efficiency of the extract.

## 4. Discussion

**Figure 11:**
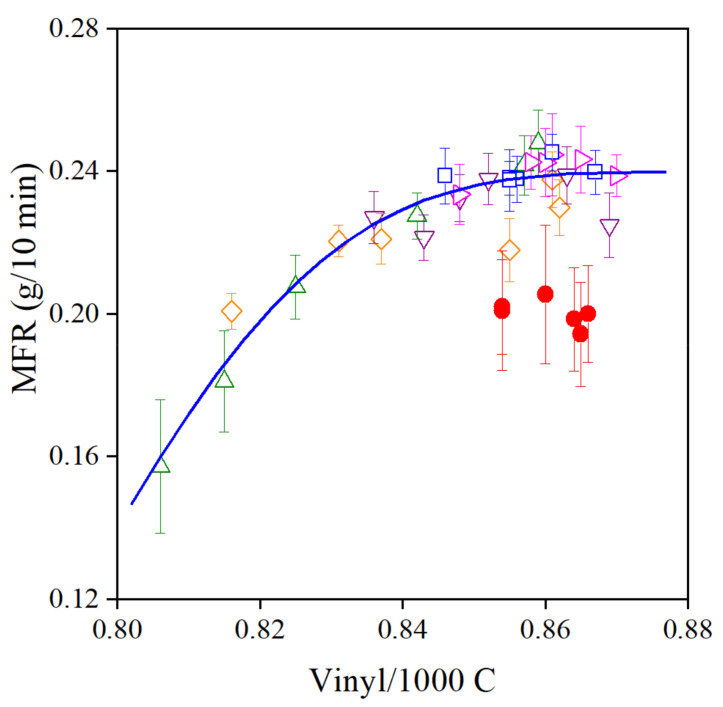
Correlation between the MFR and vinyl group content of PE containing various amounts of POM extract with or without PEPQ secondary stabilizer. Symbols: (△) 50, (◇) 100, (▽) 250, (▷) 500 and (□) 1000 ppm POM with PEPQ, (●) 1000 ppm POM without PEPQ.

The results presented in the previous sections prove that the pomegranate extract produced in our lab is an excellent processing stabilizer, but it does not practically provide any long-term stability to the polymer. The additive obviously can react with C-centered alkyl radicals with large efficiency, thus preventing the addition of the latter to the vinyl groups of the polymer. Consequently, the viscosity of the polymer does not change during processing. This statement is strongly supported by the correlation between the vinyl content and the MFR of the polymer shown in Figure 11. The increasing additive content leads to a larger number of unreacted vinyl groups and smaller viscosity. The group of full circles belong to PE samples stabilized with 1000 ppm POM alone, without the phosphorous secondary antioxidant. It is interesting to note that PEPQ has a definite effect: the two additives act synergistically, just like in the case of traditional hindered phenolic antioxidants.

In view of the large number of components in the POM extract and their complicated structure, the identification of the reason for the contradictory behavior, i.e., efficient processing stabilization and poor long-term stability, is extremely difficult, one can only speculate. Crystalline structure, high melting point, and poor solubility cannot be the reason, as shown in the previous section. The interaction of the molecules of the extract, on the other hand, probably strongly influences reactivity and efficiency.

**Scheme 2:**
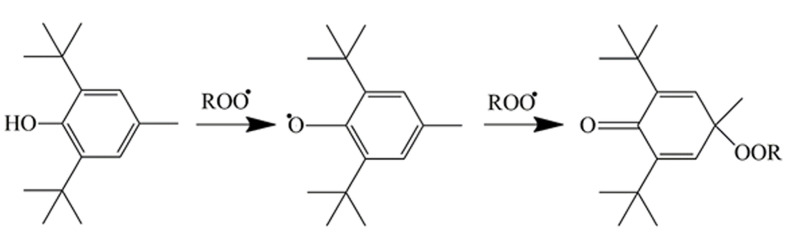
Stabilization mechanism of a traditional hindered phenolic antioxidant. Example of one of the possible reactions.

The results indicate that not physical or physical-chemical, but purely chemical reasons result in the smaller activity under oxygen-rich conditions. Alkyl radicals are extremely reactive [42]; thus, they must also react with the active phenolic hydrogens of the molecules found in the extract. Oxygen-centered radicals are less reactive [42] and thus might not compete successfully with the self-interaction of the additive molecules. Moreover, besides the donation of an active hydrogen, phenolic antioxidants enter into further reactions with each other and also with oxygen-centered radicals. Isomerization, dimerization, and the formation of chinoidal compounds are all part of the stabilizing action of phenolic antioxidants [38,43,44]. One of these reactions is shown in Scheme 2 to demonstrate the complexity of stabilization reactions. The molecules in the POM extract obviously cannot enter into such reactions or only with a very slow probability and/or rate. Delocalization of the electron forming after hydrogen abstraction from the phenolic OH groups or the lack of hydrogen atoms in the para position in sufficient numbers and with high enough reactivity might also result in the lack of stabilization efficiency under oxygen-rich conditions. Whatever the reason, it is quite obvious that POM extracts can be used for the processing stabilization of polymers, but not in applications in which large, long-term stability is needed.

## 5. Conclusions

The overview on the composition of POM extracts showed that they contain a very large number of components in various amounts. Composition depends on the genotype and source of the fruit as well as on the extraction methods used. The results of experiments carried out as a function of stabilizer concentration confirmed the excellent processing stabilization efficiency of the extract, but also the poor long-term stability of PE containing it, in accordance with previously published results. The extract is amorphous and its solubility is relatively large in the polymer; thus, these factors cannot be the reason for the poor stabilization efficiency in an oxygen-rich environment. Chemical factors, the self-interaction of the polyphenol molecules, the stability of the radicals forming after hydrogen abstraction from the phenolic OH groups, and the lack of hydrogens with the necessary activity hinder the reaction of the additive molecules with oxygen-centered radicals, thus leading to the inferior long-term stability. The POM extract can be used for the processing stabilization of polymers, but for applications requiring long-term stability. it must be combined with other natural antioxidants like flavonoids or Vitamin E.

## Figures and Tables

**Table 1:** Overview of the extraction methods (solvents) and main components of pomegranate peel extracts.

Author	Solvent	Extract		Ref.
		Components ^(a)^	Amount ^(b)^ (mg/g)	
Fisher, U.A.	MeOH/water	punicalagin (a)	15.5	[16]
		granatin B (b)	4.4	
		galloyl-HHDP-glucose (c)	3.8	
Li, R.	MeOH/EtOH/ acetone/water	punicalagin	62.0–103.6	[19]
		ellagic acid (d)	2.7–7.1	
		punicalin (e)	1.7–3.9	
Lu, J.	EtOH/water	punicalagin	39.8–120.5	[24]
Prešurić, Ž.	EtOH/water/ formic acid	punicalagin	n.a.	[25]
		granatin		
Oudane, B.	MeOH	punicalagin	105.8	[26]
Abid, M.	water EtOH, acetone	punicalagin	n.a.	[27]
		galloyl-HHDP-hexoside (f)		
		granatin B		
Feng, L.	EtAc	punicalagin	138.2	[28]
		gallic acid (g)	18.5	
		ellagic acid	12.7	
Khalil, A.A.	MeOH	punicalagin	110.0	[29]
	EtOH		96.5	
	EtAc		88.7	
Sabraoui, T.	MeOH	punicalagin	121.7–196.8	[30]
Rongai, D.	water/EtOH	punicalagin	1.6–476.0	[31]
Çam, M.	pressurized water	punicalagin	116.6	[32]
Tátraaljai, D.	acetone	punicalagin	79 ± 5.2	this work
		ellagic acid	15.3 ± 0.002	

^(a)^ The chemical structure of the components is presented in Scheme 1. ^(b)^ The amount of active component in the dry peel.

## Data Availability

Data is contained within the article.
